# Tough Love: Impact of High-Performance Work System on Employee Innovation Behavior

**DOI:** 10.3389/fpsyg.2022.919993

**Published:** 2022-06-23

**Authors:** Fuyun Zhu, Ying Gao, Xiaotun Chen

**Affiliations:** School of Economics and Management, Shaanxi University of Science and Technology, Xi’an, China

**Keywords:** high-performance work system, employee innovation behavior, challenge stress, perceived organization support, tough love

## Abstract

Based on the social exchange theory, this paper discusses the impact of high-performance work system (HPWS) on employee innovation behavior, constructs the mediating model of challenge stress and the moderated mediation model, and explores the influence mechanism of HPWS on employee innovation behavior under the management mode of combining strictness and love formed by “strictness” under the effect of challenge stress and “love” given by perceived organizational support. Through hierarchical regression analysis of 227 employees’ survey data, the results show that HPWS positively influences employee innovation behavior. Challenge stress partially mediates the above relationship, and perceived organizational support positively moderates the mediating effect of challenge stress between HPWS and employee innovation behavior.

## Introduction

In the context of digital transformation, the rapid changes in the market environment in which organizations operate, the external environment faced by organizations has become more complicated and highly uncertain, and the adjustment and remodeling of the organizational structure have become inevitable trend. High-performance work system (HPWS) is a series of coordinated HRM practices that can improve employees’ knowledge and skills and organizational benefits and make employees become the key to establishing and maintaining the core competitiveness of the organization (Datta et al., 2005). It contains a series of HRM practices, and it is a positive way to adjust the organizational structure. Under the influence of HPWS, some changes may occur when employees are stimulated by the external environment, including behavior and attitude.

Innovation is the first power to bring sustainable development to organizations. Organizations are also paying more and more attention to obtaining and maintaining their competitive advantages through innovation-driven development (Montani et al., 2017). As the basis of enterprise innovation, employees and their innovation behaviors help the organization improve its efficiency and maintain its competitive advantage (Miao and Cao, 2020). Enterprise innovation is inseparable from the employee. How to promote employee innovation has always been a hot issue concerned by the practical and academic circles. As a driving factor of employee innovation, the organizational environment has been widely recognized as related to innovation behavior. The emergence of employee innovation behavior is closely related to the innovation platform and opportunities provided by the organization. HPWS not only adapts to the needs of organizational management reform in the rapid change of the market environment but also creates good conditions for the organization to implement innovation-driven. At the same time, the existing relevant studies have confirmed that HPWS plays a part in improving the attitudes or behaviors of employees, including the ability to promote creativity and innovative behavior (Escribá-Carda et al., 2017; He et al., 2017). However, as an important influencing factor of creative behavior, the role of work stress between HPWS and employee innovation behavior has not attracted sufficient attention from the academic circles.

To further explain how HPWS affects employee innovation behavior, the mediation mechanism of the challenge stress between HPWS and employee innovation behavior is explored in this study. Currently, many empirical studies have proved the correlation between stress and employee innovation behavior. Most of the studies have shown that stress is an effective way to affect the generation of innovation behavior (Lepine et al., 2004; Han and Sun, 2019). In management practice, dealing with work stress has become a problem that modern enterprise managers must be aware of and pay attention to Hamidi and Eivazi (2010). Since Cavanaugh et al. (2000) proposed the challenge and hindrance dimensions of stressors, relevant studies have confirmed that challenge stress is positive stress that can motivate employees to study hard and promote their growth at work. The high-performance requirements set by HPWS can create challenge stress for employees. Challenge stress is conducive to activating the mind, enabling individuals to generate unconventional problem-solving methods and inspire innovation.

At the same time, the organization which implements HPWS manifests itself as the tough-love approach in management. To be more specific, the organizational management is “tough” with high expectations and will put challenge stress on employees in various aspects. For example, they will set higher performance goals for employees and require higher quality and ability. Simultaneously, as a kind of “love” that can effectively help and guide employees, perceived organizational support felt by employees could directly enhance the positive impact of challenge stress. Moreover, it can make employees feel a harmonious organizational atmosphere. This combination of “strictness” and “kindness” will make employees less worried about innovation risks and promote innovative behaviors. Therefore, this study established a moderated mediating model with perceived organizational support as the moderate variable and researched the moderator of perceived organizational support on the mediating effect of challenge stress.

**FIGURE 1:**
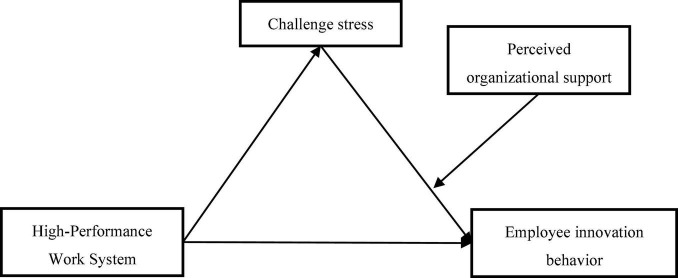
The research framework of this study.

In conclusion, supported by social exchange theory, this study examined how HPWS affects employee innovation behavior, investigated the mediation mechanism of challenge stress, and constructed a moderated mediating model with perceived organizational support as the moderating variable (as summarized in Figure 1).

## Theory and Hypotheses

### Social Exchange Theory

Homans (1958) puts forward the social exchange theory based on behaviorism from the perspective of individual needs and psychological motivation. He believes that the behavior of individuals is essentially an exchange activity with the group to obtain what they want. Blau (1964) improved and enriched Homans’ view and explained the social exchange theory from the principle of reciprocity, arguing that people are willing to exchange behaviors only when the pay and return are equal.

Social exchange theory provides a theoretical basis for explaining the impact of HPWS on employee behavior. According to the social exchange theory, there is an exchange relationship between organizations and employees. Providing employees with working conditions that meet expectations will make employees spend more at work and are willing to take positive attitudes and behaviors (Blau, 1964). Social exchange theory emphasizes the principle of reciprocity. Organizations pay more and more attention to improving organizational performance through exchange relationships with employees (Nadeem et al., 2019). The principle of reciprocity is manifested in the organizational management of HPWSs: the organization can provide employees with a series of best human resource (HR) management practices in exchange for employee feedback, including employees’ positive attitudes and positive behaviors (Miao and Cao, 2020). Supported by the social exchange theory, this paper explores the relationship between HPWS and employee innovation behaviors. Organizations that implement HPWSs provide employees with various resources and organizational support when they are under work stress. It will exchange employees’ positive attitudes and behavior feedback and create conditions for innovative behavior. Therefore, social exchange theory provides a theoretical basis for this paper to study the relationship between HPWS, employee innovation behavior, challenge stress, and perceived organizational support.

### High-Performance Work System and Employee Innovation Behavior

Generating, evolving, and implementing employees’ innovative ideas is defined as innovation behavior (Scott and Bruce, 1994). In the final analysis, organizational innovation ability comes from the innovation ability of its employees (Getz and Robinson, 2003). The construction of an organizational environment will promote the formation of positive attitudes and behaviors of employees and stimulate their autonomy (Du and Wang, 2022), and then increase the possibility of employees producing innovation behavior and other extra-role behaviors. According to social exchange theory, this study believes that there is an exchange relationship between the organization and the employees. An organization which provides its employees with the good working conditions will make the employees invest more in work and be willing to show positive attitudes and behaviors (Blau, 1964). While providing a series of best HRM practices to employees, organizations can exchange positive feedback from employees, such as their positive attitude and behavior (Kuvaas, 2007). Employees are expected to repay the HPWS provided by their organization with higher levels of personal emotions, attitudes, and behaviors (Pahos and Galanaki, 2022).

The exchange relationship between the organization and employees is hidden in HPWS, enabling employees to obtain better working conditions and more benefits. It motivates employees to invest more efforts to reward the organization. HPWS has a performance goal orientation and a systematic performance management method (Miao and Cao, 2020). Therefore, employees can feel a fair and reasonable performance incentive atmosphere, and their autonomy can be stimulated, which is easier to promote the generation and implementation of innovative behaviors. The implementation of the HPWS organizational training system is relatively more extensive and diversified, which provides employees with opportunities for continuous learning and development, improves the innovation ability of employees, and promotes the transformation of innovation results. The innovative incentives make the employees feel that the organization takes employees’ innovation seriously, which can stimulate their creativity and promote the generation and implementation of innovative behaviors (Hou and Hu, 2019). The employee incentive policies and measures in HPWS enable employees to be in a stimulating and harmonious organizational atmosphere. It will make the employees to give full play to their autonomy, to make more efforts to achieve work goals and to further catalyze the creation of innovation behaviors (Stremersch et al., 2022).

Relevant studies show that the influencing factors of employee innovation involve multiple levels of individual and organization (Montani et al., 2017), such as employee characteristics, knowledge and ability, leadership style, organizational atmosphere, and so on. According to the AMO theory, capability, motivation, and participation opportunity will affect employee behavior (Appelbaum et al., 2000). At the same time, individual creativity component theory also believes that professional knowledge, creative thinking ability, and internal task motivation are the main components of creativity. Organizations that implement HPWS provide employees with the resources, participation opportunities, and a good innovation atmosphere. These factors can improve the knowledge and skills of employees, then encourage them to increase their work involvement and stimulate their work motivation (Mashi et al., 2022). From the above discussion, it can be inferred that HPWS significantly affects employee innovation behavior. Therefore, we put forward the following hypothesis:

**H1:** HPWS positively affects employee innovation behavior.

### The Mediating Role of Challenge Stress

Work stress includes the opposite parts of challenge stress and hindrance stress. The positive and motivating stress is called challenge stress, which will make employees more enthusiastic about work and bring positive effects to employees, while hindrance stress is the contrary (Cavanaugh et al., 2000). When existing studies discuss work stress in organizations, it is generally believed that the interaction between an environmental stimulus and individual response promotes stress generation (Beehr and Newman, 1978). Therefore, the role of the organizational environment on stress cannot be ignored. Organizations that implement HPWS have high expectations for their employees, so they are strict in performance management. That is, they have higher requirements on the performance of their employees and the employees need to put more effort such as energy and resources into work to meet work performance requirements (Deepakshi and Akansha, 2019). Therefore, HPWS brings more work stress to employees under the performance-oriented management by objectives. When employees are in an organization that implements HPWS, they see the stress they face through self-regulation. HPWS sets high-performance goals for employees and provides them with positive practices such as training and knowledge sharing, which motivates employees’ intrinsic motivation. This is when employees classify the stress they face as challenge stress, which positively affects their attitudes and behaviors (Kristin et al., 2020).

As mentioned above, HPWS has also been proved to positively affect employees. Therefore, we assume the following views:

**H2:** HPWS positively affects challenge stress.

The influence of stress on employee behavior has been widely recognized. Challenge stress is closely related to employee behavior. Challenge stress can activate the individual’s cognitive system and bring about a positive “problem-solving” coping plan (Christopher et al., 2020). Employees’ innovative behavior itself is a process of solving real problems. A meta-analysis of ambidextrous stress also showed that challenge stress was positively correlated with positive factors such as employee engagement, job satisfaction, job performance, and organizational citizenship behavior (Dik et al., 2012; Zhang et al., 2015). At the same time, challenge stress can help employees integrate into their work, improve their sense of work meaning and professional identity, and motivate staff to increase job involvement. The positive factors can stimulate employee creativity promotion and affect employees’ innovation performance (Han and Sun, 2019). Moreover, it plays a pivotal part in promoting the generation, evolution, and practice of employee innovation behavior. Therefore, we assume the following:

**H3:** Challenge stress positively affects employee innovation behavior.

At the same time, according to the above assumptions, challenge stress can positively affect employee innovation behavior. HPWS can also bring positive effects to employees through a transmission mechanism. Therefore, combining H2 and H3, the following hypotheses are proposed:

**H4:** The effect of HPWS on employee innovation behavior will be mediated by challenge stress.

### Moderating Effects of Perceived Organizational Support

Difficult and achievable goals are the most effective for employees. Otherwise, if the difficulty of goals cannot be translated into actual work motivation, employees will lose confidence in completing challenging tasks. Therefore, providing employees with the support they need can eliminate their concerns and reduce their over-perception of the difficulty of actual work goals. It will help employees overcome the fear of facing high-performance challenging goals, which is an efficient way for employees to carry out an important guarantee for innovative work (Zhu et al., 2021). The extent to which employees perceive that the organization cares about their well-being and cares about their contributions is known as perceived organizational support (Eisenberger et al., 1986). As an organizational level of support, perceived organizational support is a factor employees rely on when facing work stress (Zacher and Winter, 2011). In the face of stress, when employees feel strong recognition, encouragement, and support from the organization, they will be more motivated and enthusiastic about increasing their work engagement (Rich et al., 2010), thus generating behaviors conducive to organizational development. It will enhance the positive impact of challenge stress and conducive to the generation of individual innovation behaviors and the transformation of innovation achievements (Wallace et al., 2009). Therefore, we assume the following hypothesis:

**H5:** Perceived organizational support moderates the effect of challenge stress on employee innovation behavior.

With the joint action of challenge stress and perceived organizational support, the organization implementing HPWS forms a management mode of “tough-love approach.” HPWS sets strict performance requirements and goals for employees, brings challenge stress to employees, and motivates employees to generate innovative thinking and innovative behavior. Based on social exchange theory, when employees feel cared for and supported by the organization, it is more likely to show positive feedback behaviors, increase work engagement and intrinsic motivation, and thus increase innovative behaviors. Therefore, we consider that perceived organizational support can moderate the mediating effect of challenge stress between HPWS and employee innovation behavior. The mediator of challenge stress between HPWS and employee innovation behavior will also be moderated by perceived organizational support. To summarize, this study further proposes a moderated mediation model. In other words, challenge stress mediates the effect of HPWS on employee innovation behavior, but the strength of the mediating effect depends on the perceived organizational support level. To verify this, we hypothesize the following views:

**H6:** Perceived organizational support moderates the mediating effect of challenge stress on the impact of HPWS on employee innovation behavior.

## Materials and Methods

### Sample and Procedure

We conducted a questionnaire survey of employees of Chinese companies with work experience by email. We invited employees of companies that our team has cooperated with for a long time in research work, graduate alumni of our school engaged in management positions, and other employees of their companies to participate in this survey. As the participants were distributed in different cities in China, the data was collected through an online survey. The three authors contacted the HRs departments of the surveyed companies and graduate alumni in management positions through telephone and online contact. They encouraged their employees to participate in our survey. We explained the purpose of our research in the questionnaire and promised to participate in the survey anonymously and only for academic research, which guarantees the confidentiality of respondents’ information. Podsakoff et al. (2003) believe that temporal separation is an effective procedural remedy for common method bias. In order to reduce the threat of common method biases (Podsakoff et al., 2003), the online questionnaire was distributed in two waves (1 month apart). A total of 270 marked matching questionnaires were distributed. HPWS, employee innovation behavior, and perceived organizational support were measured in Wave 1. The control variables and challenge stress were measured 1 month later in Wave 2. In the end, 227 valid and matched complete questionnaires were collected, and the response rate for this study was 84.1%. A total of 47.6% of the participants were male. Those aged 35 and under accounted for 62.1%. Participants worked in diverse occupations, including production, marketing, technology, administration, and management positions.

### Measures

In order to make the research more effective, the scales used in this study are all derived from published results. We used a 5-point Likert-type scale for the measurement of all variables.

#### High-Performance Work System

Zhang et al.’s (2013) 12-item HPWS scale was used to measure HPWS. Sample items include “My company’s performance appraisal system focuses on performance and work results” and “My company organizes special training on the performance appraisal system for employees” (Cronbach alpha = 0.839).

#### Challenge Stress

Six items from Cavanaugh et al. (2000) were adapted to measure challenge stress. Sample items include “I have a lot of projects or tasks” to measure challenge stress (Cronbach alpha = 0.700).

#### Perceived Organizational Support

Eight items from Eisenberger et al. (1986) were adapted to measure perceived organizational support. Sample items include “When I have difficulties, my company will help me” and “My company is very concerned about me” (Cronbach alpha = 0.814).

#### Employee Innovation Behavior

A 6-item scale from Scott and Bruce (1994) was used to measure employee innovation behavior. Sample items include “At work, I will look for or apply new technologies, new procedures and new methods” and “At work, I often generate some creative ideas and innovative ideas” (Cronbach alpha = 0.734).

#### Control Variables

Aim at reducing the interference of other unrelated variables on the research results: gender, age, education, position, and position level were taken as the control variables in this study.

### Confirmatory Factor Analysis and Common Method Biases

Since the scales used in this study are all mature scales, we conducted confirmatory factor analysis (CFA) for five key variables to test the discriminative validity through AMOS23.0. The results in Table 1 show that the four-factor CFA model’s indicators are within the reference range (χ^2^ = 587.596, *df* = 454, TLI = 0.921, CFI = 0.928, RMSEA = 0.036, SRMR = 0.056). Moreover, the four-factor model fit is better than other alternative competition models. Therefore, these four variables will be used in the subsequent data analysis.

**TABLE 1:** Results of confirmatory factor analysis.

Model	χ^2^	*df*	TLI	CFI	RMSEA	SRMR
One-factor model	877.056	464	0.759	0.775	0.063	0.069
Two-factor model combining HPWS, challenge stress and perceived organizational support	814.820	463	0.795	0.808	0.058	0.067
Three-factor model combining challenge stress and perceived organizational support	659.607	457	0.880	0.890	0.044	0.060
Four-factor model	587.596	454	0.921	0.928	0.036	0.056

*TLI, Tucker–Lewis index; CFI, comparative fit index; RMSEA, root mean square error of approximation; SRMR, standardized root mean residual.*

In addition, Harman’s single-factor test was used in this study to test for common method bias. The results showed that seven factors with eigenvalues greater than one were extracted, and the unrotated first factor explained 26.15% of the variance, which was less than 40%. Therefore, there is no serious common method bias in this study.

## Results

### Descriptive Analyses

The results of descriptive analyses are shown in Table 2. The correlation between the variables is analyzed, and the correlation coefficient indicates that the variables in this study are all significantly correlated (*p* < 0.01). The means, standard deviations, and correlation coefficient of each variable are shown in Table 2. The results showed that HPWS was positively correlated with employee innovation behavior (*r* = 0.501, *p* < 0.01) and challenge stress (*r* = 0.429, *p* < 0.01). Moreover, challenge stress was positively correlated with employee innovation behavior (*r* = 0.470, *p* < 0.01). These results provided a preparatory test for the hypothesis testing of this study.

**TABLE 2:** Results of descriptive analyses.

Variable	*M*	SD	1	2	3	4	5	6	7	8	9
1. Gender	1.52	0.501	1								
2. Age	2.19	1.221	−0.192**	1							
3. Education	2.71	0.869	0.026	−0.306**	1						
4. Position	3.38	1.356	0.234**	−0.308**	0.068	1					
5. Position level	1.78	0.971	−0.198**	0.532**	−0.291**	−0.283**	1				
6. HPWS	2.23	0.505	0.041	−0.286**	0.118	0.125	−0.138*	1			
7. EIB	2.21	0.529	0.089	−0.318**	0.063	0.199**	−0.270**	0.501**	1		
8. CS	2.25	0.511	0.135*	−0.433**	0.198**	0.174**	−0.316**	0.429**	0.470**	1	
9. POS	2.39	0.586	0.075	−0.455**	0.146	0.227**	−0.304**	0.599**	0.523**	0.493**	1

N = 227, *p < 0.05, **p < 0.01.

*HPWS stands for high performance work system; EIB stands for employee innovation behavior; CS represents challenge stress; POS represents perceived organizational support.*

### Hypotheses Testing

#### Mediating Effect Testing

To test the mediating effect hypotheses in this study, we adopted a series of hierarchical multiple regression analyses. The hierarchical regression test results are shown in Table 3.

**TABLE 3:** Results of the hierarchical multiple regression analysis.

Variable	Challenge stress	Employee innovation behavior				
		
	Model 1	Model 2	Model 3	Model 4	Model 5	Model 6
Gender	0.048	0.006	−0.020	−0.007	0.001	0.010
Age	−0.254***	−0.109	−0.095	−0.042	0.003	0.004
Education	0.047	−0.072	−0.078	−0.084	−0.070	−0.050
Position	0.009	0.070	0.082	0.068	0.052	0.017
Position level	−0.110	−0.151*	−0.096	−0.122	−0.082	−0.050
HPWS	0.332***	0.449***		0.361***		
Challenge stress			0.402***	0.265***	0.271***	−0.083
Perceived organizational support					0.364***	−0.045
Challenge stress × perceived organizational support						0.407*
*R* ^2^	0.304	0.311	0.255	0.36	0.345	0.357
*F*	16.037***	16.516***	12.561***	17.474***	16.442***	15.129***
						

*p < 0.05, ***p < 0.001.

First, in Model 2, after controlling the effects of gender, age, education, position, and position level, the significant positive effect of HPWS on employee innovation behavior was revealed (β = 0.449, *p* < 0.001). Therefore, H1 was verified.

Second, in model 1, there was a significant positive impact of HPWS on challenge stress (β = 0.332, *p* < 0.001). Thus, H2 was supported. Model 3 shows that challenge stress significantly positively impacted employee innovation behavior (β = 0.402, *p* < 0.001), and H3 was verified.

Third, based on model 2, add mediating variable challenge stress to form Model 4. Model 4 shows HPWS was still significantly positive influence employee innovation behavior (β = 0.361, *p* < 0.001), and the direct effect of HPWS on employee innovation behavior is abated. In other words, challenge stress partially mediated the impact of HPWS on employee innovation behavior. Thus, H4 was supported.

To further verify whether the mediation effect exists, the PROCESS program developed by Hayes (2013) was used for the Bootstrap test. The Bootstrap test results are shown in Table 4. The indirect effect of challenge stress was significant (*r* = 0.092, *BootLCI* [0.032, 0.166]), while the direct effect was weakened but still at a significant level (*r* = 0.378, *BootLCI* [0.253, 0.503]). The Bootstrap test results were consistent with the regression analysis results.

**TABLE 4:** Bootstrap test results of mediation effect.

	Effect	SE	Bootstrap 95% CI
Direct effect: HPWS → challenge stress → employee innovation behavior	0.378	0.063	[0.253, 0.503]
Indirect effect: HPWS → challenge stress → employee innovation behavior	0.092	0.035	[0.032, 0.166]
			

#### Moderating Effect Testing

In order to test the moderating effect, we first standardized the variables and then performed the corresponding data analysis. Model 6 in Table 3 shows that the interaction coefficient between challenge stress and perceived organizational support was significant and positive (*b* = 0.407, *p* < 0.05). Perceived organizational support positively moderates the effect of challenge stress on employee innovation behavior. In other words, when perceived organizational support is strong rather than weak, the effect will be stronger. So H5 was supported.

In order to test the moderated mediation effect, Model 14 in the PROCESS program was adopted in this study to perform a Bootstrap test on the moderated mediation hypothesis. The test results are shown in Table 5. Under the moderation of high perceived organizational support, challenge stress has a significant indirect effect on the impact of HPWS on employee innovateon behavior. At the same time, the confidence interval did not include 0 (*r* = 0.102, *BootLCI* [0.041, 0.176]). Under the moderation of low perceived organizational support, the indirect effect of HPWS on employee innovation behavior through challenge stress did not reach a significant level, and the confidence interval included 0 (*r* = 0.034, *BootLCI* [−0.038, 0.111]). Finally, the significance of the indirect effect difference was tested, and it was found that the indirect effect difference reached a significant level, and the confidence interval did not include 0 (*r* = 0.068, *BootLCI* [0.065, 0.079]). These results revealed that the mediating effect of challenge stress would be stronger when perceived organizational support is strong rather than weak. Therefore, H6 was supported.

**TABLE 5:** Bootstrap test results of moderated mediation.

Moderator	Indirect influence coefficient	Bootstrap 95% CI
Low perceived organizational support	0.034	[−0.038, 0.111]
High perceived organizational support	0.102	[0.041, 0.176]
Difference	0.068	[0.065, 0.079]

*CI, confidence interval.*

**FIGURE 2:**
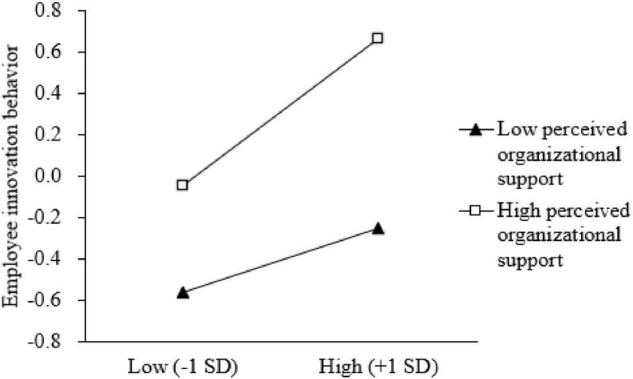
Moderating diagram of organizational support on mediating effect.

In addition, in order to better understand the moderating effect of perceived organizational support on the mediating effect of challenge stress, we divided the participants into high and low groups according to the mean of perceived organizational support plus or minus 1 SD to investigate the influence of challenge stress on employee innovation behavior at different levels of perceived organizational support. The slope diagram is shown in Figure 2. The results showed that when the level of perceived organizational support was low, the mediating effect of challenge stress in predicting employee innovation behavior was not significant; when the level of perceived organizational support was high, the challenge stress significantly positively predicted the employee innovation behavior. As the challenge stress increased, the higher perceived organizational support, the easier it was for employees to produce innovative behaviors under challenge stress.

## Discussion

As HPWS is considered an essential factor that affects employee attitudes and behaviors, the existing literature is mainly based on AMO theory to discuss the role of motivation, self-efficacy, organizational learning, etc., on employee innovation behavior under the influence of HPWS (Escribá-Carda et al., 2017; Luo and Qian, 2018). However, work stress as a critical factor affecting innovation has been neglected. From the perspective of work stress and supported by social exchange theory, this research tested the positive effect of HPWS on employee innovation behavior, the mediator of challenge stress, the moderator of perceived organizational support, and the moderated mediating model. The empirical results showed that HPWS significantly positively affected employee innovation behavior, challenge stress partly mediated the effect of HPWS on employee innovation behavior, and perceived organizational support positively moderated the mediating effect of HPWS on employee innovation behavior through challenge stress.

### Theoretical Implications

First, this research enriches employee innovation behavior studies in the context of HPWS. Distinctly, employee innovation behavior plays a role in organizational development. Previous studies have shown that HPWS can significantly affect employee attitudes and behaviors (Pahos and Galanaki, 2022), including innovative behaviors (He et al., 2017). However, due to the different research perspectives and research mechanisms, the existing studies are controversial about the relationship between HPWS and employee innovation behavior (Sun and Wang, 2016). This study verified that HPWS positively predicts employee innovation behavior, which will further enrich the empirical research on HPWS and employee innovation. The results also further clarify the impact of the organizational environment on employee innovation behavior.

Second, this study enriches the related research on organizational environment and innovative behavior from the perspective of stress. The existing literature discussed the influence mechanism of HPWSs on employee innovation and creativity based on motivation (Luo and Qian, 2018) and capital perspectives (Miao and Cao, 2020). Previous studies have ignored the transmission effect of stress between HPWS and innovative behavior. Supported by social exchange theory, the effect of HPWS on the mechanism of employee innovation behavior is investigated in this study. It proved that challenge stress partially mediated the influence of HPWS on employee innovation behavior. This paper uses challenge stress as the transmission mechanism to explain the transmission path of HPWS to employee innovation behavior more comprehensively. This research also enriches the research on dependent variables and independent variables of challenge stress and further verifies the interaction between stress and environment.

Finally, this study further clarifies the boundary of the effect of challenge stress on employee innovation behavior. Some scholars have found that perceived organizational support can enhance the positive effect of challenge stress (Zhu et al., 2021). This study uses perceived organizational support as a moderator variable to verify the moderating role of perceived organizational support as the mediating effect of “love” given by the organization to the challenge stress. We combine HPWS, challenge stress, perceived organizational support, and employee innovation behavior into one model and build a management model of “tough-love,” which enriches the theoretical explanation of the impact of HPWS on employee innovation behavior. At the same time, it also provides a new perspective for studying the interaction between stress and the environment.

### Practical Implications

First, with the rapid changes in the social environment, the transformation of organizational management has become an inevitable trend to conform to the development of The Times (Kumar et al., 2016). This research could help organizations actively explore building HPWS to establish a reasonable and fair performance evaluation system and rewards and punishment system. Moreover, this research could help organizations set up effective information sharing and communication between staff, subordinates, leadership, and departments. Also, it could help them to establish a people-oriented corporate culture, strengthen the humanistic care and let employees work in the necessary resources and conditions to inspire employees to invest more effort in work and promote the improvement of employees’ work attitudes and behaviors, and then promote employee innovation behavior increases. It will lay the foundation for the organization to maintain its core competitive advantage and achieve sustainable development.

Second, this study could help managers attach importance to employee innovation and the construction of innovative staff teams. In increasingly fierce market competition, organizations must recognize the importance of stimulating employee innovation to establish and maintain their competitive advantage. Therefore, organizational managers should be committed to establishing a reasonable innovation incentive and reward mechanism. That will provide an institutional guarantee for employees’ innovation, form an orderly innovation management model, and then promote the improvement of organizational innovation performance. The organization also should establish an innovative talent training system and be attentive to the cultivation of innovative talents, as well as carry out the innovation project training to improve staff innovation ability and increase investment in innovation of money or other resources to ensure that meet the demand of employees’ innovation. It will reduce staff innovation behavior barriers and create a favorable innovation atmosphere to improve the innovation efficiency and performance of employees.

Finally, organization managers should not only control the work stress of employees but also provide encouragement and support. In job design, organizational managers can take some measures to provide employees challenge stress, such as set strict requirements on employees and place high expectations on them to encourage employees to fully explore their potential and create conditions for the generation of innovation behaviors. In addition, when organization managers notice that employees face high-strength work stress, they should go as far as possible to give their staff encouragement and support to create a loving organizational atmosphere and let employees feel love from the organization. That will improve employees’ sense of belonging to the organization and motivate them to put more effort into the work and let stress into motive force, then catalyze innovation behavior. According to these, it will bring more innovation and performance output to the organization and enable the personal development of employees and the improvement of organizational efficiency to achieve mutual benefit and win-win results.

### Limitations and Future Research

First, we adopted a static research method instead of a dynamic research method due to the limitations of research conditions. However, employee innovation behavior is not fixed, and work stress can also change from time to time (Wang et al., 2022). In future studies, the combination of static and dynamic investigation methods, such as the daily diary method, should be considered as much as possible to verify the hypothesis more effectively. Second, the mediator of hindrance stress is not considered in this paper. The next time, we will explore the influence of other types of organizational environments on work stress and further explore the influence of two-dimensional stressors in different directions.

## Data Availability Statement

The raw data supporting the conclusions of this article will be made available by the authors, without undue reservation.

## Ethics Statement

Ethical review and approval were not required for the study on human participants in accordance with the local legislation and institutional requirements. Written informed consent for participation was not required for this study in accordance with the national legislation and the institutional requirements.

## Author Contributions

FZ, YG, and XC contributed to conception and design of the study. YG and XC collated and analyzed the data. YG wrote the first draft of the manuscript. FZ and XC wrote sections of the manuscript. All authors contributed to manuscript revision, read, and approved the submitted version.

## Conflict of Interest

The authors declare that the research was conducted in the absence of any commercial or financial relationships that could be construed as a potential conflict of interest.

## Publisher’s Note

All claims expressed in this article are solely those of the authors and do not necessarily represent those of their affiliated organizations, or those of the publisher, the editors and the reviewers. Any product that may be evaluated in this article, or claim that may be made by its manufacturer, is not guaranteed or endorsed by the publisher.
